# Effects of maternal hypoxia on placental levels of oxidative stress markers in COMT^-/-^ and C57 mice

**DOI:** 10.1186/1753-6561-6-S4-P10

**Published:** 2012-07-09

**Authors:** DF Thambiraj, CF Rueda-Clausen, J Stanley, R Poudel, S Davidge, P Baker

**Affiliations:** 1 Royal College of Surgeons in Ireland, Ireland; 2 Department of Obstetrics and Gynecology, University of Alberta, Canada

## Introduction

In preeclampsia, it is believed that widespread endothelial dysfunction leads to reduced placental perfusion and increased oxidative stress. Oxidative stress is the accumulation of reactive oxygen species (ROS) such as superoxide (O2-), nitric oxide (NO), and peroxynitrite (ONOO-). Our lab has previously established a colony of transgenic mice that do not express catechol-O-methyl transferase (COMT-/-). COMT produces 2-methoxyestradiol (2-ME), a potent vasodilator that is normally increased in pregnancy. Pregnant COMT-/- mice exhibit a phenotype similar to the one observed in preeclampsia. We hypothesize that COMT-/- mice have a decreased tolerance to prenatal hypoxic insults, characterized by an increase in placental oxidative stress when compared to control (C57) mice exposed to similar conditions.

## Methods

From gestational day 10.5 to 18.5, COMT-/- and C57 control mice were randomized to either normoxic (21% O_2_) or hypoxic (10.5% O_2_) conditions. Placentas were dissected at day 18.5, cryopreserved, sliced, and stained for imaging via fluorescent microscopy. Dihydroethidium (DHE) stains were used to assess superoxide levels, while Nitrotyrosine stains were used to assess peroxynitrite levels. Liver sections were used as a standardizing control. Data is presented as mean and standard error of the mean (SEM) or median and interquartile range (IQR) depending on data distribution. The amount of luminescence relative to the liver control were plotted and then analyzed via two-way ANOVA and a Bonferroni post-hoc test.

## Results

No significant differences in placental levels of superoxide were observed among experimental groups (effect of genotype p=0.17, hypoxia p=0.82 and interaction p=0.54). Under normoxic conditions, placental levels of peroxynitrite were comparable between COMT-/- and C57 mice. Under hypoxic conditions however, placental tissues from COMT-/- hypoxic (p<0.05), but not C57 hypoxic mice (p=0.40), exhibited a significant increase in peroxynitrite relative to tissues from normoxic animals with the same genotype.

## Conclusions

There appears to be a synergistic interaction between genotype and hypoxia in the development of increased placental oxidative stress. The observed increase of peroxynitrite in placental tissues from COMT-/- hypoxic mice may be the result of increased superoxide production, increased bioavailability of nitric oxide, or a combination of both.

